# Arginine methylation dysfunction increased risk of acute coronary syndrome in coronary artery disease population: A case-control study

**DOI:** 10.1097/MD.0000000000006074

**Published:** 2017-02-17

**Authors:** Shengyu Zhang, Shuyang Zhang, Hongyun Wang, Wei Wu, Yicong Ye

**Affiliations:** a Department of Internal Medicine; b Department of Cardiology; c Clinical Pharmacology Research Center, Peking Union Medical College Hospital, Chinese Academy of Medical Sciences, Beijing, China.

**Keywords:** acute coronary syndrome, asymmetric dimethylarginine, methylation

## Abstract

The plasma levels of asymmetric dimethylarginine (ADMA) had been proved to be an independent cardiovascular risk factor. Few studies involved the entire arginine methylation dysfunction. This study was designed to investigate whether arginine methylation dysfunction is associated with acute coronary syndrome risk in coronary artery disease population.

In total 298 patients undergoing coronary angiography because of chest pain with the diagnosis of stable angina pectoris or acute coronary syndrome from February 2013 to June 2014 were included. Plasma levels of free arginine, citrulline, ornithine, and the methylated form of arginine, ADMA, and symmetric dimethylarginine (SDMA) were measured with high-performance liquid chromatography coupled with tandem mass spectrometry. We examined the relationship between arginine metabolism-related amino acids or arginine methylation index (AMI, defined as ratio of [arginine + citrulline + ornithine]/[ADMA + SDMA]) and acute coronary events.

We found that plasma ADMA levels were similar in the stable angina pectoris group and the acute coronary syndrome group (*P* = 0.88); the AMI differed significantly between 2 groups (*P* < 0.001). Multivariate logistic regression demonstrated that AMI was an independent risk factor of acute coronary events in patients with coronary artery disease (OR = 0.975, 95% confidence interval 0.956–0.993; *P* = 0.008).

Our study suggested that ADMA levels were very similar in the stable angina and acute coronary syndrome patients; AMI might be an independent risk factor of acute coronary events in coronary artery disease population.

## 1 Introduction

Asymmetric dimethylarginine (ADMA) is an endogenous inhibitor of all 3 isoforms of nitric oxide synthase (NOS), which originates from degradation of methylated arginine (Arg) residues in the course of physiologic protein turnover. It has been shown that ADMA plasma levels are elevated in diseases which are linked to an impairment of the NO-pathway and endothelial dysfunction such as hypertension, hypercholesterolemia, type 2 diabetes mellitus, hyperhomocysteinemia, and chronic renal failure.^[1,2]^ Especially, ADMA was found to be an independent cardiovascular risk factor of coronary artery disease (CAD) and in clinical trials and meta-analysis.^[3–6]^

**Figure 1:**
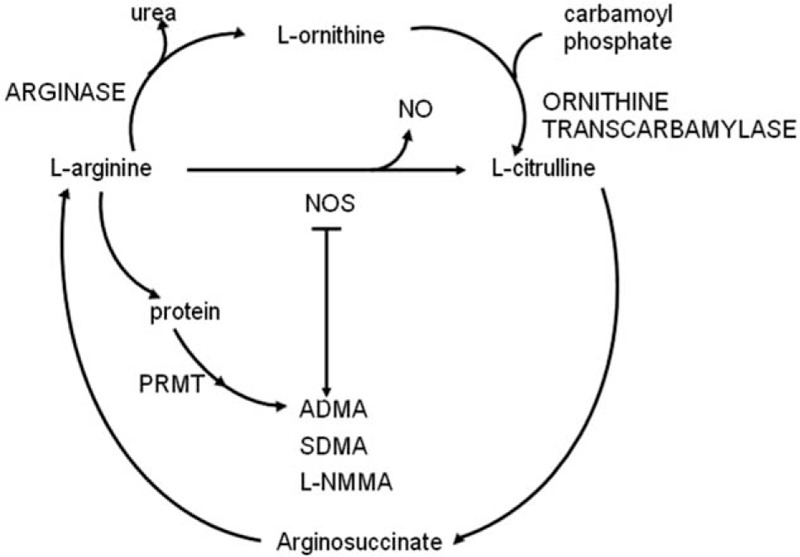
Schematic illustrations of pathways for arginine metabolism. The main metabolism pathway includes several important enzymes: NOS that converts Arg to Cit and NO; arginase that converts Arg to Orn; ornithine transcarbamylase that converts Orn to Cit and further to Arg via arginosuccinate; PRMT that transfers methyl to Arg residues in protein; DDAH that hydrolyses ADMA to Cit and dimethylamine. The first 3 enzymes are main part of the normal pathway, whereas the last 2 comprised the methylation pathway. ADMA = asymmetric dimethylarginine, Arg = arginine, Cit = citrulline, DDAH = dimethylarginine dimethylaminohydrolase, NMMA = N^G^-monomethylarginine, NO = nitric oxide, NOS = nitric oxide synthase, Orn = ornithine, PRMT = protein arginine methyltransferase, SDMA = symmetric dimethylarginine.

The main Arg metabolism pathway includes several important enzymes (Fig. 1). Since plasma levels of ADMA and SDMA are much more higher than NMMA, we used (Arg + Cit + Orn) for the unmethylated metabolites and (ADMA + SDMA) for the methylated metabolites in the pathway. The ratio of (Arg + Cit + Orn)/(ADMA + SDMA) may indicate global arginine methylation dysfunction.

Nowadays few studies involved the entire arginine methylation dysfunction in atherosclerotic diseases. So we designed this case-control study to investigate whether arginine methylation dysfunction is associated with acute cardiac event in coronary artery disease population.

## 2 Methods

### 2.1 Patients

Two separate experienced cardiologists evaluated 412 consecutive coronary angiograms. Finally, 298 patients undergoing coronary angiography because of chest pain with the diagnosis of stable angina pectoris (SAP) or acute coronary syndrome (ACS) from February 2013 to June 2014 were included in this study. According to history and result of coronary angiograms, patients were divided into 2 groups: SAP group (defined as ≥50% stenosis in major coronary arteries^[7]^) and ACS group (diagnosed according to ACC/AHA guidelines^[8,9]^). Exclusion criteria included the following: renal dysfunction (serum creatinine >1.5 mg/dL), hepatic or thyroid diseases, acute/chronic infection, malignant tumors, cerebrovascular disease, history of ACS, symptoms, or signs suggestive of congestive heart failure, and other myocardiopathy. The traditional cardiovascular risk factors included age, sex, systemic hypertension, hypercholesterolemia, smoking, and diabetes mellitus. Systemic hypertension was diagnosed if blood pressure was ≥140/90 mm Hg on 2 occasions or if the patients were taking antihypertensive drugs. Hypercholesterolemia was defined as fasting total cholesterol level ≥200 mg/dL or if the patients were already taking lipid-lowering agents. Diabetes mellitus was diagnosed if there was a fasting glucose level ≥126 mg/dL and/or plasma glucose level of ≥200 mg/dL 2 hours after glucose administration, or if the patient was taking oral hypoglycemic agents or receiving insulin injection therapy for blood glucose control at the present time. Acute/chronic infection status was determined through temperature taking, physical examination, and basic laboratory tests (i.e., complete blood count, chest x-ray).

Before coronary angiogram, written consents were obtained and peripheral venous blood samples were drawn for measurement of complete blood count, creatinine, ALT, lipid profile (including total cholesterol [TC], low-density lipoprotein cholesterol [LDL-C], high-density lipoprotein cholesterol [HDL-C], and triglyceride [TG]), HbA1c and high sensitive C-reaction protein (hsCRP). We estimated the creatinine clearance rate (CCr) with Cockcroft–Gault formula. For results of ultrasonic cardiograph and myocardial perfusion imaging, we referred to the medical records.

The severity of coronary atherosclerosis was summarized by “coronary atherosclerotic score” (CAS) developed by Azar et al^[10]^ by 2 separate experienced cardiologists. In brief, the coronary artery tree was divided into 9 segments: the left main coronary artery; the proximal, middle, and distal left anterior descending artery; the proximal and distal circumflex artery; the proximal and middle right coronary artery; and the posterior descending artery. Each of these segments was scored from 0 to 3 depending on the most severe diameter stenosis according to the following system: 0 = normal, 1 = stenosis between 1% and 49%, 2 = stenosis between 50% and 99%, 3 = total stenosis, with each of the segments distal to the occlusion arbitrarily given a score of 1. The CAS was generated as the sum of the scores in all segments.

### 2.2 Plasma ADMA assay

Fasting blood was drawn under standardized conditions. Samples were immediately processed and stored at –80°C until analysis. Plasma levels of ADMA, SDMA, Arg, Cit, and Orn were measured by high-performance liquid chromatography coupled with tandem mass spectrometry (HPLC-MS/MS).^[11,12]^ Briefly, 100 μL plasma was mixed with 25 μL of internal standard 15N-L-lysine (50 μmol/L) and 500 μL of acetonitrile, and then vortexed for 30S. After centrifugation at 10,000* g* for 10 minutes, 500 μL of the supernatant was collected and evaporated to dryness under a gentle stream of nitrogen at room temperature. The dryness was reconstituted with 120 μL of pure water and filtered through a 0.2 μm nylon micro-spin filter tube (Alltech, Deerfield, IL) at a centrifugation of 10,000*g* for 1 minutes. The filtration was collected and chromatographed on an Atlantis dC_18_ column (4.6 × 100 mm, 5 μm; Waters, Milford, MA) with water (0.1% formic acid)-acetonitrile (0.1% formic acid) (95:5, *v/v*) as the mobile phase. The flow rate was 0.25 mL/min and the injection volume was 10 μL. The detection was performed on the API 3000 triple-quadrupole mass spectrometer (AB Sciex, Foster City, CA) using electrospray positive ionization (working setting: nebulizer pressure 30 psi, drying temperature 350, drying gas 7 L/min). Multiple reactions monitoring (MRM) scan mode was applied to monitor the target analytes based on the following mass transitions: ADMA, 203.1 → 46.2; SDMA, 203.1 → 172.4; Arg, 175.1 → 70.0; Cit, 176.1 → 70.0; Orn, 133.1 → 70.0; 15N-Lyscine (internal standard), 148.1 → 85.0, respectively. The HPLC-MS/MS method was validated via standard solution over the concentration range of 0.25 to 4 μmol/L for ADMA and SDMA, and 25 to 400 μmol/L for Arg, Cit, and Orn, respectively. Inter- and intra-day precision for the target analytes were all within 15% and the accuracy was within 85% to 115%. The limit of quantitation was 0.25 μmol/L for ADMA and SDMA, and 25 μmol/L for Arg, Cit, and Orn, respectively.

### 2.3 Statistical analysis

Data were analyzed with the SPSS 17.0 for Windows (SPSS Inc., Chicago, IL). Continuous variables were presented as means ± standard deviation (SD) and categorical variables as frequency and percentage. Comparisons between the 2 groups were performed using Student *t* tests for continuous variables and the χ^2^ tests or Fisher exact tests for categorical data. The multivariate logistic regression analysis was used to evaluate the independent risk factors of ACS. The odds ratios (OR) and 95% confidence intervals (CI) were calculated. A 2-tailed *P*-value of <0.05 was considered significant.

This study was carried out with the approval of ethics committee of Peking Union Medical College Hospital, and they monitored for rationality and safety.

## 3 Results

### 3.1 Baseline characteristics

In total 298 patients were enrolled in this study, including 98 patients in the SAP group and 200 patients in the ACS group. There were no significant differences between 2 groups concerning age, sex, body mass index (BMI), mean artery pressure (MAP), CCr, TG, TC, LDL-C, HDL-C, left ventricle ejection fraction (LVEF), and other cardiovascular risk factors including hypertension, hyperlipidemia, diabetes, smoking habit, and family history of CAD, even secondary prevention medicine-use (Table 1). Compared to the SAP group, only the percentage of hsCRP elevation (>10 mg/L) in the ACS group was significantly higher (4.0% vs 31.0%, *P* < 0.001).

**Table 1:**
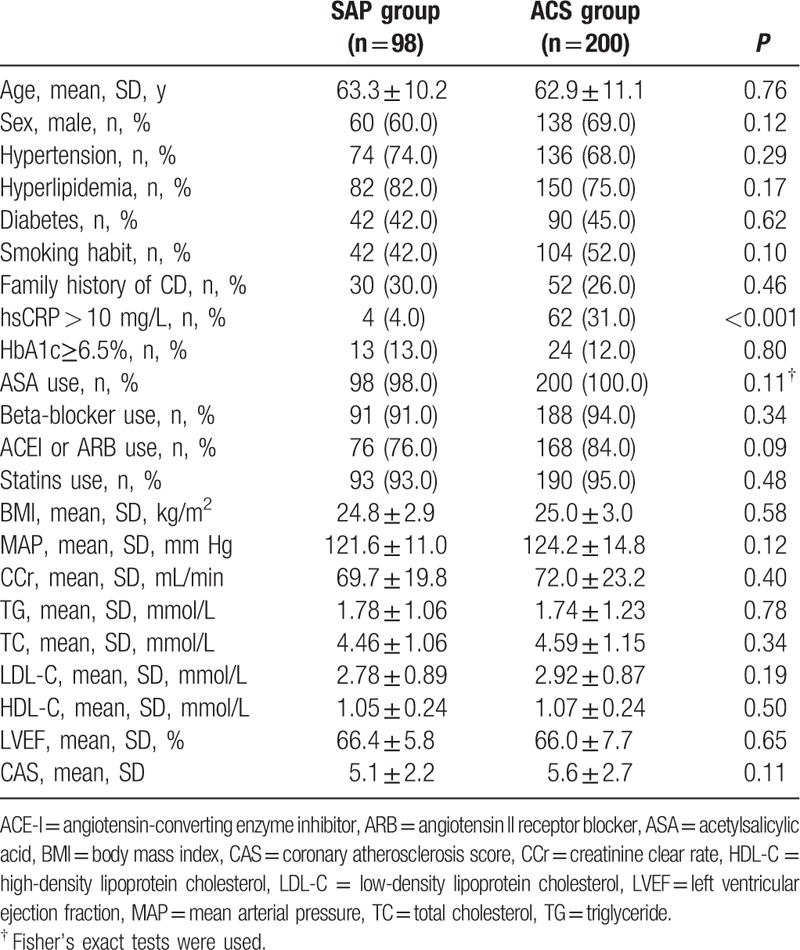
Baseline characteristics of SAP and ACS groups.

### 3.2 The levels of arginine metabolism-related items

As shown in Table 2, plasma ADMA levels in both groups were very similar (0.311 ± 0.054 vs 0.312 ± 0.054 μmol/L, *P* = 0.88). The plasma concentrations of Arg in the ACS group were significantly lower than the SAP group (10.2 ± 7.7 vs 13.6 ± 9.3 μmol/L, *P* = 0.001). Similarly, concentrations of Cit and Orn in the ACS group were also significantly lower than the SAP group (both *P* < 0.001). The AMI in the SAP group was significantly higher than that of the ACS group (110.6 ± 27.9 vs 93.4 ± 22.6, *P* < 0.001).

**Table 2:**
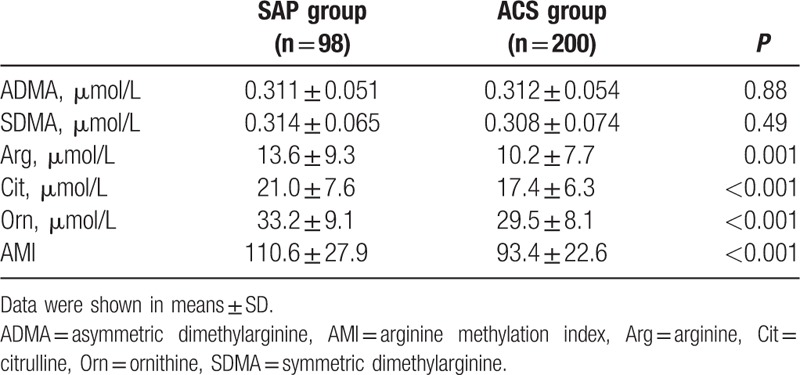
The levels of arginine metabolism-related items in SAP and ACS groups.

### 3.3 Multivariate regression analysis of risk factors for ACS

To access whether the AMI-associated independently with ACS, the multivariate stepwise logistic regression model was calculated, and all established risk factors or risk markers for CAD and secondary prevention medicine-use were included. AMI and hsCRP were identified as the independent protector or risk factor for ACS (OR = 0.975, 95% CI 0.956–0.993, *P* = 0.008; OR = 1.258, 95% CI 1.100–1.439, *P* = 0.001, respectively).

## 4 Discussion

ADMA is synthesized when arginine residues in proteins are methylated by arginine methyltransferases and is partly eliminated from through renal filtration, which interacts with the NO pathway and leads to endothelial dysfunction. Therefore, many studies have been conducted to establish the association between the serum ADMA level and the CAD risk. However, limited studies focused on the entire arginine methylation metabolism. So we designed the first case-control study to explore the global arginine methylation dysfunction in CAD patients.

Our first important finding was that the plasma AD22MA levels in both SAP and ACS groups were very similar. Frøbert et al^[13]^ compared plasma concentration of ADMA in patients of SAP, non-ST segment elevation, and ST segment elevation myocardial infarction, and found little difference existed. However, Krempl et al^[14]^ demonstrated that ADMA levels in unstable angina pectoris patients were significantly higher than SAP patients, in which “multivessel disease” was considered to reflect complication of vascular lesions. However, the cases in both studies were relatively limited and did not fully consider the complication and severity of vascular lesions, which proven to be related with ADMA levels,^[15]^ that was higher ADMA could result from more severe vascular lesion. The CAS in both groups had no significant difference in our study, which indicated that ADMA levels should be similar between SAP and ACS patients after eliminating bias from complicated vascular lesions.

Further, we first reported that plasma levels of Arg, Cit, and Orn in the ACS group were significantly lower than the SAP group. These small amino acids were important in arginine metabolism, which were related to nitric oxide generation and arginine cycle. Under normal diet, Arg exogenous intake was relatively fixed; only 5% to 15% plasma Arg was synthesized in the body (mainly in kidney),^[16]^ and the rest came from body protein degradation; so increased Arg catabolism may be the reason for plasma level declining. Many studies demonstrated that under status of hypertension, hyperlipidemia, and diabetes, the activity of arginase in the plasma and vascular tissue would increase,^[17–19]^ which might result from oxidative stress. The upregulation of arginase is of central importance for reduced NO bioavailability due to competition for the substrate Arg between arginase and endothelial NO synthase (eNOS). Also, evidence suggests a putative role of oxidative stress in CAD, especially in ACS, which was believed to be a predictive factor for CAD and even could predict major adverse cardiovascular events.^[20,21]^ Aukrust et al^[22]^ also suggested elevated oxidative stress concomitant with inflammatory markers in ACS. This implied arginase activity might also increase in CAD and ACS conditions, which was proven in vitro,^[23]^ and would further induce low bioavailability of arginine. Low substrate bioavailability for NOS and arginase would explain low Cit and Orn concentrations. But whether arginase activity is even significantly higher in ACS than SAP, which will answer the question why levels of Arg, Cit, and Orn were even lower in the ACS patients, needs further investigation.

We also first demonstrated that AMI in the SAP group was significantly higher than that of the ACS group. Despite of diet intake influence, plasma Arg hemostasis was achieved largely via protein degradation and Arg catabolism.^[15]^ So the AMI was partially determined by the Arg residues methylation status of protein. Oxidative stress, native or oxidized LDL, angiotensin II, eNOS inhibition/uncoupling and inflammation played a pivotal role in Arg residues methylation by managing PRMT/DDAH expression^[24–27]^, and meanwhile increased arginase activity reduced Arg bioavailability, both of which might contribute to lower AMI in ACS. Studies had demonstrated Arg metabolites were associated with atherosclerosis progression,^[15,28,29]^ and even associated with atherosclerotic plaque burden and fragility.^[30]^ Notsu et al^[31]^ suggested that the Arg/ADMA ratio may be a sensitive marker for atherosclerosis; Tang et al^[1]^ demonstrated that Arg/(Cit + Orn) (global arginine bioavailability ratio, GABR) might serve as a more comprehensive concept of reduced NO synthetic capacity and diminished GABR were associated with both development of significantly obstructive atherosclerotic CAD and heightened long-term risk for major adverse cardiovascular events. In this study, we used AMI as an index for metabolism dysfunction and found it was also an independent predictor of acute coronary events in patients of CAD.

The unavailability of easily accessible and rapid analytical methods to quantify these small amino acid molecules has been one obstacle for using Arg metabolites as risk markers. The HPLC-MS/MS has been shown to be a useful, relatively fast, and reliable method for determination of plasma levels of these small molecules with its advantage over ELISA assay.^[11,32]^ In the future, arginine metabolic amino acids may become additional biochemical parameters for clinical routine to identify those patients at higher risk for future cardiovascular events.

There were some limitations in this study. First, our study was a case-control study and sample size was relatively small. Confirmation in larger perspective cohort may be mandatory. In addition, subjects were enrolled from population referred for coronary angiography, so selection bias was inevitable for cases from a single medical center, for different admission rates might exist for SAP and ACS patients. Lastly, we considered the influence of complicate coronary lesions on ADMA levels, but neglected that of systemic atherosclerosis.

To conclude, this case-control study suggested that ADMA levels were very similar in SAP and ACS patients; lower AMI served as an independent risk factor of acute coronary syndrome for patients of coronary artery disease.
